# Life Satisfaction and Suicidal Ideation Among Chinese College Students During the Recurrent Outbreak of COVID-19: A Moderated Mediation Model

**DOI:** 10.3389/fpsyt.2022.937511

**Published:** 2022-07-11

**Authors:** Zhijun Yu, Haidong Liu, Baojuan Ye, Chunyan Tang, Dawu Huang, Lu Liu

**Affiliations:** ^1^Center of Mental Health Education and Research, School of Psychology, Jiangxi Normal University, Nanchang, China; ^2^Health Education and Counseling Center, Jiangxi Health Vocational College, Nanchang, China; ^3^Physical Education College, Jiangxi Normal University, Nanchang, China

**Keywords:** life satisfaction, suicidal ideation, depression, gratitude, COVID-19, Chinese college students

## Abstract

The present study examined a moderated mediation model between life satisfaction and suicidal ideation among Chinese college students during the recurrent outbreak of COVID-19. Seven hundred and ninety college students participated in the study and completed questionnaires on life satisfaction, suicidal ideation, depression, and gratitude. Findings indicated that (1) Depression played a partial mediating role between life satisfaction and suicidal ideation. Life satisfaction was not only directly affected suicidal ideation, but also indirectly affected suicidal ideation through the mediating effect of college students' depression; (2) Gratitude played a moderating role in the effect of life satisfaction on depression, and the link between life satisfaction and suicide ideation was only significant for those with higher gratitude. This study provides practical implications for the prevention of suicidal behavior among Chinese college students during the recurrent outbreak of COVID-19.

## Introduction

In March 2020, the World Health Organization (WHO) declared COVID-19 as a global pandemic (1). In order to prevent the further spread of the pandemic, people were forced to maintain social distance. While keeping social distance is an important measure to control the spread of COVID-19, it may affect people's mental health (2). Studies suggest that social distance may increase people's feelings of loneliness and hopelessness, which may lead to suicide (3). A longitudinal study in Germany found that the 12-month suicide ideation of German college students increased from 26.6% in 2019 to 60% in 2020, that is to say, the proportion of college students suffering from suicidal ideation in 2020 was twice as high as in previous years (4). In China, the study found that suicidal ideation among college students has been on the rise from 8.5% in February 2020 to 14.3% in June 2020 (5). So, a possible reason for the increase in suicide rates may be the isolation measures during the COVID-19 pandemic. With the increasing popularity of internet devices, people can gather a wide variety of information through the internet, and individuals at risk of suicidal behavior often approach suicide by searching the internet for information and news about self-harm and suicidal behavior, especially during adolescence (6). The World Health Organization regards reducing the incidence of non-fatal suicidal behavior and suicide mortality as the focus of suicide prevention and intervention work (7). Therefore, in the Internet era, we need to pay more attention to individual suicidal ideation to prevent occurrence of suicidal behavior. Therefore, it is essential to explore the influencing factors of people's suicidal ideation during public health emergencies which facilitates the advancement of suicide prevention as well as pandemic control.

### Life Satisfaction and Suicidal Ideation

Suicide is the leading cause of death among people aged 15–34 in China (8). 47.2% of the abnormal deaths among college students in China are suicide (9). Suicidal ideation refers to those who have lost the desire to live but have not yet caused physical injury (10). During the recurrent outbreak of COVID-19, suicidal ideation may be higher among college students due to isolation and social distancing. Therefore, it is necessary to understand the influencing factors and mechanisms of suicidal ideation in college students.

Life satisfaction refers to an individual's stable and universal overall evaluation of life conditions (11). Research showed that the pandemic affected life satisfaction (12, 13). The coronavirus threatens people's safety and desire for survival, affecting their quality of life (13). As a result, people are less satisfied with their lives during COVID-19. Life satisfaction was significantly negatively correlated with suicidal ideation (14), and life dissatisfaction was an important cause of suicide (15). To better explore the relevant factors of suicidal ideation in college students during the pandemic, it is very important to study the relationship between life satisfaction and suicidal ideation.

### Depression as a Mediator

Depression is a key indicator for diagnosing an individual's mental health (16), and usually refers to persistent negative emotional experiences in people's lives, such as oppressive, anxiety, sadness, pain, etc. (17). Individuals with depression often experience sleep disturbance, loss of appetite, and in severe cases, suicidal ideation (18). Depression seriously threatens the physical and mental development of adolescents (19), especially college students in their late adolescence (17). A study shows that depression due to COVID-19 is prevalent among Chinese college students (20) and the combined prevalence of elevated clinical depressive symptoms in adolescents during the COVID-19 pandemic is estimated to be 30.6% (21). Individuals with lower life satisfaction are more inclined to adopt negative coping styles in daily life, experience more negative emotions, and are more likely to be depressed (22, 23). Also, according to the research, temperaments greatly impact psychological distress and suicidality. Among them, the suicide attempt rate of severe depression is as high as 50%, which shows that depression is very harmful to individual psychology (24). At the same time, typical individual risk factors for suicidal ideation include psychiatric disorders, especially depression (25). Depressed individuals are prone to have suicide ideation due to low mood, pessimism, and world-weariness, and suicidal ideation is commonly seen in people with depression (26–28). Thus, we posit the following hypothesis:

**Hypothesis 1**. Depression will mediate the relationship between life satisfaction and suicidal ideation.

### Gratitude as a Moderator

Gratitude refers to an emotional trait in which an individual responds to the help of others with gratitude, thereby enabling himself to obtain a positive experience or result, which can have a positive impact on the individual's psychological development (29). At present, most of the research in the field of gratitude has focused on the relationship between gratitude and positive emotions such as life satisfaction and wellbeing (30, 31), but some researchers have found that the trait of gratitude is also closely related to the negative emotions of individuals (32, 33). According to the extended construction theory of gratitude, individuals with high gratitude traits pay more attention to the construction of interpersonal relationships, and use their interpersonal resources to solve problems, thereby reducing negative emotions that affect them, such as depression (34). Lin (35) find that gratitude is one of the important protective factors for adolescents' mental health, and it is significantly and negatively associated with depression among college students. Thus, not only does life satisfaction reduce a person's risk of depression, but also gratitude traits reduce a person's risk of depression, both of which are protective factors against depression. According to the protective-protective factor model (36), the presence of one protective factor enhances the effect of another protective factor (37). According to the model, the effect of protective factors (life satisfaction) on depression in college students may be greater for individuals with high protective factors (gratitude). Specifically, the alleviating effect of high life satisfaction on depression is stronger when individuals have higher gratitude, while the alleviating effect of high life satisfaction on depression is weaker in individuals with lower gratitude. Thus, we posit the following hypothesis:

**Hypothesis 2**. Gratitude will moderate the relationship between life satisfaction and depression.

### The Present Study

**Figure 1:**
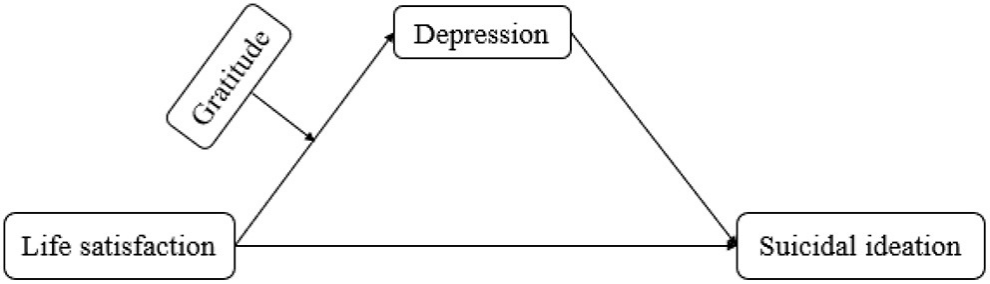
The moderated mediation model.

Taken together, the current study had two aims. First, we tested whether depression mediated the relationship between life satisfaction and suicidal ideation among Chinese college students during the recurrent outbreak of COVID-19. Second, we examined whether gratitude moderated the associations between life satisfaction and depression during the recurrent outbreak of COVID-19 (Figure 1).

## Methods

### Participants

This study was approved by the Ethics Committee of the School of Psychology, Jiangxi Normal University. Convenience sampling strategies were used. Eight hundred and nineteen questionnaires were distributed, and 790 valid questionnaires (284 males, *M*_age_ = 20.83, *SD*_age_ = 1.60) were finally collected, with a recovery rate of 96.5%. In this study, the exclusion criteria for invalid questionnaires were (1) the total score of the camouflage subscale of the Suicidal Ideation Scale ≥ 4, (2) regular responses (i.e., the same option being selected repeatedly or answer in the pattern of 1, 2, 3, 4, 5), and (3) too short a response time (<3 min). Among the 790 participants, 102 were freshmen, 260 were sophomores, 237 were juniors and 191 were seniors. All participants provided informed consent and completed the survey voluntarily.

### Measures

#### Life Satisfaction Scale

The Chinese version (38) of the Life Satisfaction Scale (39) was used to measure life satisfaction. There is one dimension with five items (e.g., “*In most ways my life is close to my ideal*”). Each item is scored on a seven-point Likert scale ranging from 1 (*strongly disagree*) to 7 (*strongly agree*). The average score of the five items was calculated, with higher average scores representing higher life satisfaction. In the present study, the McDonald's ω coefficient of the Life Satisfaction Scale was 0.93.

#### Gratitude Scale

The Chinese version (29) of the Gratitude Questionnaire (40) was used to measure gratitude. Participants rated six items (e.g., “*When I set myself an objective, I continue until I achieve it*”) on a seven-point scale ranging from 1 (*strongly disagree*) to 7 (*strongly agree*). The average score of the six items (two items are reverse scored) was calculated, with higher scores reflecting greater gratitude. In the present study, the McDonald's ω coefficient of the Gratitude Questionnaire was 0.78.

#### Depression Scale

Depression was measured by the nine-item Patient Health Questionnaire (41). The Chinese version was revised by Bian et al. (42). This scale consists of nine items (e.g., “*Feeling down depressed or hopeless*”). Each item was rated on a four-point scale (0= *not at all to* 3= *almost every day*). The average score of the nine items was calculated, with higher scores reflecting more severe depression. In the present study, the McDonald's ω coefficient of the Patient Health Questionnaire was 0.94.

#### Suicidal Ideation Scale

Suicidal ideation was measured by the Self-Rating Idea of Suicide Scale (43). The scale was specially developed under the background of Chinese culture. This scale consists of 26 items (e.g., “*I wanted to end my life*”), including 4 subscales: hopelessness (12 items), sleep (4 items), optimism (5 items), and camouflage (5 items). The camouflage subscale was used to determine whether the participant's responses were truthful. If the total score of the camouflage subscale ≥ 4, the test was invalid. Therefore, the scores of the camouflage subscale were not included in the total score and the study used the mean scores of the three subscales of hopelessness, sleep, and optimism (43), with higher scores representing higher suicidal ideation. The scale is scored from 0 to 1, with 0 representing no and 1 representing yes. In the present study, the McDonald's ω coefficient of the Self-Rating Idea of Suicide Scale was 0.86.

### Procedure

The survey was hosted on Wenjuan Web (Shanghai Zhongyan International Science and Technology, Shanghai, China; https://www.wenjuan.com/) from January 2 to 12, 2022. Most Chinese college students were still in school preparing for their final exams during this period, so participants were not in a closed-school period. Specifically, we first created an online survey, and then generated a QR code to facilitate publishing. The QR code was then sent to WeChat and QQ groups (i.e., online social media platforms) where interested participants could participate in the study. Participants completed the survey anonymously to collect information on gender, age, life satisfaction, suicidal ideation, gratitude, and depression. In this study, participants provided informed consent. In this study, all responses were anonymous. There was no compensation for participating in this study, and the participants participated entirely voluntarily. The study was approved by the ethics committee of the first author's university.

## Results

### Preliminary Analyses

The means and Pearson correlations among the study variables are presented in Table 1. Life satisfaction was negatively correlated with depression (*r* = −0.22, *p* < 0.001) and suicidal ideation (*r* = −0.41, *p* < 0.001), and positively correlated with gratitude (*r* = 0.23, *p* < 0.001). Gratitude was negatively correlated with depression (*r* = −0.35, *p* < 0.001) and suicidal ideation (*r* = −0.31, *p* < 0.001). Additionally, depression was positively correlated with suicidal ideation (*r* = 0.68, *p* < 0.001).

**Table 1:** Descriptive statistics.

	***M***	***SD***	**1**	**2**	**3**	**4**
1 Life satisfaction	4.67	1.25	1			
2 Gratitude	5.02	0.84	0.23***	1		
3 Depression	1.06	0.77	−0.22***	−0.35***	1	
4 Suicidal ideation	0.29	0.23	−0.41***	−0.31***	0.68***	1

N = 790; ***p < 0.001.

### Testing for Mediation Effect

Hypothesis 1 was tested with Equation 4 of the PROCESS macro (44). As Table 2 Equation 1 (suicidal ideation) showed, life satisfaction was negatively related to suicidal ideation [β = −0.41, *t* = −12.65, 95%CI (−0.48, −0.35), *p* < 0.001]. According to Equation 2 (depression) and Equation 3 (suicidal ideation), life satisfaction was significant negatively related to depression [β = −0.23, *t* = −6.48, 95%CI (−0.29, −0.16), *p* < 0.001] and significant negatively related to suicidal ideation [β = −0.27, *t* = −10.84, 95%CI (−0.32, −0.22), *p* < 0.001]. Depression was positively associated with suicidal ideation [β = 0.61, *t* = 24.36, 95%CI (0.56, 0.66), *p* < 0.001]. Thus, hypothesis 1 was supported, and depression partially mediated the relationship between life satisfaction and suicidal ideation.

**Table 2:** Testing the mediation effect of life satisfaction on suicidal ideation.

**Predictors**	**Equation 1**		**Equation 2**		**Equation 3**		**Equation 4**	
	**(suicidal ideation)**		**(depression)**		**(suicidal ideation)**		**(depression)**	
	**β (95%CI)**	***t***	**β (95%CI)**	***t***	**β (95%CI)**	***t***	**β (95%CI)**	***t***
Age	−0.01 (−0.05, 0.03)	−0.38	−0.0004 (−0.04, 0.04)	−0.02	−0.008 (−0.04, 0.02)	−0.49	−0.001 (−0.04, 0.04)	−0.07
Gender	−0.03 (−0.19, 0.07)	−0.88	−0.08 (−0.23, 0.06)	−1.14	−0.009 (−0.11, 0.09)	−0.18	−0.07 (−0.21, 0.06)	−1.03
Life satisfaction	−0.41 (−0.48, −0.35)	−12.65***	−0.23 (−0.29, −0.16)	−6.48***	−0.27 (−0.32, −0.22)	−10.84***	−0.15 (−0.21, −0.08)	−4.38***
Depression					0.61 (0.56, 0.66)	24.36***		
Gratitude							−0.33 (−0.39, −0.26)	−9.62***
Life satisfaction × gratitude							−0.12 (−0.18, −0.06)	−4.05***
*R* ^2^	0.17		0.05		0.53		0.16	
*F*	53.36***		14.09***		218.52***		29.82***	

N = 790; ***p < 0.001.

### Moderated Mediation Effect Analysis

The moderated mediation model was tested with Model 7 of the SPSS macro-PROCESS (44). The results were shown in Equation 4 of Table 2. The product of life satisfaction and gratitude (the interaction term) was significantly associated with depression [β = −0.12, *t* = −4.05, 95%CI (−0.18, −0.06), *p* < 0.001], suggesting that gratitude could moderate the relationship between life satisfaction and depression. Specifically, gratitude could moderate the first half of the indirect pathway. Hypothesis 2 was supported.

**Figure 2:**
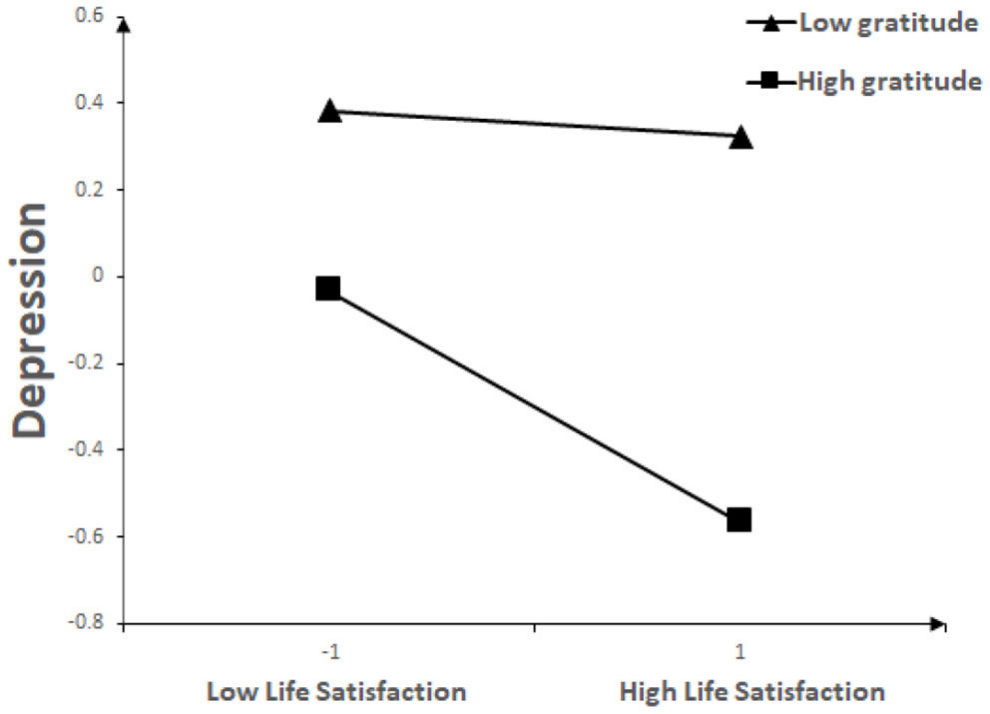
Interaction between life satisfaction and gratitude on depression.

For descriptive purposes, we plotted and explored life satisfaction against depression, separately for low and high gratitude. The interaction effect was visually plotted in Figure 2. Simple slope tests showed that for college students with high gratitude, life satisfaction was significantly associated with depression (β = −0.27, *t* = −6.06, *p* < 0.001). As for college students with low gratitude, life satisfaction had no significant effect on depression (β = −0.03, *t* = −0.65, *p* > 0.05).

## Discussion

Through a survey of 790 Chinese college students, this study found that life satisfaction was significantly negatively associated with college students' suicidal ideation during the recurrent outbreak of COVID-19. After verifying the direct link, this study constructed and tested a moderated mediation model to explore the mechanism of life satisfaction on suicidal ideation. This study further found that depression played a partial mediating role between life satisfaction and suicidal ideation in college students. Gratitude moderated the relationship between life satisfaction and depression.

### Life Satisfaction and Suicidal Ideation

A significant negative association between life satisfaction and suicidal ideation was found which was consistent with the findings of non-pandemic period studies (45, 46). Individuals with low life satisfaction are more likely to have feelings of hopelessness, which can easily lead individuals to have suicidal thoughts (47). This suggests that we should pay attention to college students' life satisfaction, both during pandemic and non-pandemic periods, and enhance college students' life satisfaction to reduce their suicidal ideation.

### The Mediating Role of Depression

Based on verifying the relationship between life satisfaction and suicidal ideation of college students, this study also deeply explored the mediating effect of depression on life satisfaction and college students' suicidal ideation, that is, life satisfaction affected college students' suicidal ideation through depression during the recurrent outbreak of COVID-19, which supported the hypothesis 1. The results of the study showed a significant negative association between life satisfaction and depression, which was consistent with pre-pandemic studies (22). Compared to the pre-pandemic period, people were less satisfied with their current living situation due to fear of COVID-19 and various control measures, as well as economic and psychological stress during the pandemic, leading to a decrease in life satisfaction (12). Low life satisfaction leads to more negative affect, which affects individuals' mental health and thus increases their risk of depression. A study showed that the depression incidence rate caused by the COVID-19 increased by about 27.6% in 2020 (48). And the depression detection rate in the Chinese general population during the COVID-19 pandemic outbreak was as high as 53.5% (49). Therefore, we suggest that the life satisfaction of college students should be taken seriously whether or not during the COVID-19 pandemic.

Depression is one of the risk factors for suicide. The persistent negative effect brought by depression makes individuals prone to thoughts of wanting to end their lives and escape the pain of the situation at hand. As a result, individuals with depression are more likely to tolerate and accept suicide (50). According to clinical studies, 15% of patients with major depression have a high risk of suicide (51). The findings of this paper also confirm a significant correlation between depression and suicide ideation, which was consistent with studies conducted during non-pandemic periods (52, 53). A study in Japan found a 16% increase in monthly suicide rates during the second wave of the COVID-19 pandemic (July to October 2020) (54). Thus, we need to be especially concerned about the depression status of college students, especially during the COVID-19 pandemic, which is very important for suicide prevention among college students. During the pandemic, individuals face challenges to their mental health due to quarantines and other measures. This study enriches previous studies based on university students who were under pandemic control measures and has important implications for the prevention of suicidal ideation among university students during the COVID-19 pandemic.

### The Moderating Role of Gratitude

This study also examined the moderating role of gratitude between life satisfaction and depression. The present study found that gratitude was significantly and negatively associated with depression, which was consistent with the findings of the pre-pandemic COVID-19 study (55). Gratitude plays an important role in post-traumatic growth, especially during the pandemic period, and gratitude has many physical and psychological benefits for individuals, which can lead to a decrease in depression (56, 57). Therefore, activities to express gratitude can be actively pursued, which may help to cope with some of the psychological problems during the COVID-19 pandemic. This study also found that gratitude has a mitigating effect on the relationship between life satisfaction and depression during the recurrent outbreak of COVID-19, that is, when the individual's gratitude was low, the effect of life satisfaction on depression was not significant; but when the individual's gratitude was high, the inhibitory effect of life satisfaction on depression was significant. However, the pre-pandemic studies of COVID-19 did not examine the moderating role of gratitude between life satisfaction and depression. According to the gratitude coping hypothesis, individuals with high gratitude respond positively to stresses and difficulties; on the contrary, individuals with low gratitude adopt more negative attitudes to deal with the hardships in life, resulting in negative behaviors such as avoidance, and thus adversely affect individual physical and mental health (58). Fredrickson (59) believes that individuals with high gratitude are good at dealing with interpersonal relationships and can get help and support from interpersonal resources in the face of difficulties, thus reducing irritability and depression. During the pandemic, grateful individuals may be better able to cope with COVID-19-related stress and are more likely to seek support from others (60). These findings have implications for the prevention of depression in college students in the future. Therefore, it is important to improve the gratitude and life satisfaction of college students during both pandemic and non-pandemic periods. According to our research results, college students who have low life satisfaction and low gratitude were most likely to be depressed, so attention should be paid to improving their life satisfaction and gratitude.

### Limitations and Future Directions

This study had some limitations. First, this study did not consider other control variables, such as other demographic variables (except age and gender) and mental state variables that may have an impact on the study results. In the future, these control variables should be taken into account. Second, this study adopted a cross-sectional design, which cannot infer the causal relationship between variables in a strict sense. In the future, a longitudinal tracking experimental design can be considered to further examine the relationship between variables. Third, this study adopted a self-report questionnaire. Surveys may be affected by social favorability, especially for qualities that are highly socially desirable, such as gratitude. In the future, measures with less social favorability can be considered, such as the use of forced-choice questionnaires. Finally, considering the current study was conducted among Chinese college students during the recurrent outbreak of COVID-19, its generalizability was limited and future studies should be conducted in a more diverse sample to verify the validity of the current study in other cultural contexts.

Despite these limitations, this study also has some practical implications. First, according to the research results, low life satisfaction will not only increase the depression of college students but also affect their suicidal ideation of college students. Therefore, colleges and relevant departments should pay attention to the quality of life of college students, to better promote the improvement of life satisfaction of college students and reduce the risk of depression and suicide. Second, considering that depression plays a mediating role between life satisfaction and suicidal ideation in college students, attention should be paid to the depression status of college students and their mental health. Finally, gratitude can moderate the impact of college students' life satisfaction on depression, and college students are also a critical period for the formation and development of individual gratitude (61). Therefore, it is necessary to pay attention to cultivating the gratitude characteristics of college students and to use effective intervention methods (such as “conscious focus on blessings”) to improve their gratitude awareness. Empirical research has shown that gratitude interventions can improve people's gratitude and life satisfaction (62).

## Conclusion

In summary, although further research was needed, this study represented an important step in exploring how life satisfaction may be related to suicidal ideation among Chinese college students during the recurrent outbreak of COVID-19. This study showed that depression played a partial mediating role between life satisfaction and suicidal ideation. Life satisfaction was not only directly and positively related to suicidal ideation, but also indirectly affected suicidal ideation through the mediating effect of college students' depression. Moreover, gratitude played a moderating role in the effect of life satisfaction on depression, and the relationship between life satisfaction and suicidal ideation became stronger to college students with high gratitude.

## Data Availability Statement

The raw data supporting the conclusions of this article will be made available by the authors, without undue reservation.

## Ethics Statement

The studies involving human participants were reviewed and approved by Research Ethics Committee of Jiangxi Normal University. The patients/participants provided their written informed consent to participate in this study.

## Author Contributions

ZY, HL, and BY designed the study. ZY collected the data. ZY, HL, BY, and CT analyzed the data and conceptualized the models. CT supervised the project. DH and LL made important modifications to the paper. All authors have seen, wrote, approved the manuscript, and revised the manuscript.

## Funding

This study was supported by the National Natural Science Foundation of China (72164018), National Social Science Fund Project (BFA200065), Jiangxi Social Science Foundation Project (21JY13), Jiangxi'Key Research Base Project of Humanities and Social Sciences (JD20068), and Science and Technology Research Project of Jiangxi' Department of Education (GJJ200306).

## Conflict of Interest

The authors declare that the research was conducted in the absence of any commercial or financial relationships that could be construed as a potential conflict of interest.

## Publisher's Note

All claims expressed in this article are solely those of the authors and do not necessarily represent those of their affiliated organizations, or those of the publisher, the editors and the reviewers. Any product that may be evaluated in this article, or claim that may be made by its manufacturer, is not guaranteed or endorsed by the publisher.
